# Anti-Inflammatory and Survival Benefits of Dipeptidyl Peptidase 4 Inhibitors Among Patients with Gout, T2DM Patients and Chronic Kidney Disease

**DOI:** 10.1055/a-2565-7419

**Published:** 2025-04-29

**Authors:** Shachaf Shiber, Amir Sharabi, Irit Ayalon, Eviatar Naamany, Alon Grossman, Yair Molad

**Affiliations:** 1 36632 Rheumatology, Rabin Medical Center, Petah Tikva, Israel; 2 58408 Tel Aviv University Faculty of Medicine, Tel Aviv, Israel

**Keywords:** DPP4i, Gout, Chronic kidney disease, CRP, Diabetes mellitus type 2

## Abstract

**Introduction:**

Gout and type 2 diabetes mellitus (T2DM) often coexist and are associated with chronic kidney disease (CKD) and increased mortality. Dipeptidyl peptidase-4 (DPP-4) inhibitors, commonly used in T2DM, may offer additional benefits, such as reducing inflammation and uric acid levels. This study aimed to assess the impact of DPP-4 inhibitors on gout flare frequency, serum uric acid (sUA) levels, and survival in patients with gout, T2DM, and CKD.

**Methods:**

A cross-sectional, retrospective, longitudinal study was conducted over 6 years between 2016 – 2022, including patients with gout and T2DM from the largest healthcare provider in Israel. Patients were divided into treatment and control groups based on DPP4-inhibitor status treatment. The primary outcome was the number of gout arthritis attacks over 1 year, reflected by the number of emergency room visits. Secondary outcomes included mean serum high-sensitive C-reactive protein (hs-CRP) levels and survival rates over the study period.

**Results:**

DPP-4 inhibitor treatment significantly reduced sUA levels (5.2±1.3 mg/dL vs. 5.9±2.2 mg/dL, p=0.05) and hs-CRP levels (0.50±0.19 mg/dL, p<0.001). Kaplan-Meier survival analysis suggested a trend towards improved survival in the DPP-4 inhibitor group (HR=0.834, 95% CI: 0.6–1.04, p=0.05), particularly among patients with chronic kidney disease (CKD), although without statistical significance. The emergency room visits due to gout attacks were fewer in the DPP-4 inhibitor group, although this difference did not achieve statistical significance.

**Conclusion:**

DPP-4 inhibitors may offer benefits beyond glycemic control in T2DM and gout, including reduced sUA and hs-CRP levels and improved survival in CKD patients. Larger, randomized trials are warranted to explore these potential benefits.

## Introduction

Gout is a common autoinflammatory arthritis induced by the deposition of monosodium-urate crystals (MSUC) in the joint, characterized by acute recurrent arthritis attacks with the subsequent development of tophi 1 2 . The single most important risk factor for developing gout is excess uric acid in the extracellular fluid, which results in the precipitation of MSUC 3 4 . Hyperuricemia leads to the deposition of MSUC in the renal tract. It appears to be an independent risk factor for the development of chronic kidney disease (CKD) and cardiovascular events, particularly in patients with type 2 diabetes mellitus (T2DM) 5 6 7 8 . Interestingly, a high serum uric acid (sUA) level correlates with higher insulin resistance 9 . In a retrospective study, Jaffe DH et al. 10 found that approximately 30% of patients with CKD who were newly diagnosed with gout were linked to T2DM during a 5-year follow-up period.

Incretin-based therapies (e. g., dipeptidyl peptidase 4 [DPP-4] inhibitors and glucagon-like peptide 1 [GLP-1] receptor agonists) affect glucose control through several mechanisms, including enhancement of glucose-dependent insulin secretion, slowing gastric emptying, and reduction of postprandial glucagon and food intake. 11 12

Sitagliptin, saxagliptin, linagliptin, and alogliptin are DPP-4 inhibitors approved for treating T2DM since 2006 13 .

Several studies with DPP4 inhibitors 14 15 have evaluated the potential of DPP4 inhibitors as an immune-modulating agent and as a treatment for chronic allograft dysfunction following lung transplantation. In addition, experimental studies 16 17 18 19 revealed a decreased level of pro-inflammatory cytokines such as tumor necrosis factor-α, interleukin (IL)-6, IL-17, and cluster of differentiation 163 (CD-163), as well as increased levels of anti-inflammatory cytokines such as IL-10, and transforming growth factor β.

The current study aimed to determine the incidence of gout attacks over six years in patients with gout and T2DM treated or not with DPP-4 inhibitors. We also measured the changes in serum hs-CRP levels throughout the study period.

## Methods

### Study design settings

This cross-sectional retrospective, longitudinal study was conducted on Clalit Health Service (CHS) patients, the largest of four recognized healthcare providers in Israel. CHS has approximately 4,217,000 insured citizens of all ages, representing 40% of the national population, including>60% of adults older than 65 20 . The study population was derived from a CHS database of Dan County (Gush Dan), including over 1,200,000 patients. The CHS database includes longitudinal data computerized and integrated from the central laboratory, pharmacy (including date of prescription, quantity, and time of medication dispensed), in-patient and out-patient visits at doctors, hospitalizations, and sociodemographics. All data is linked at the patient’s unique national identity number level. Death records, including the date of death, were retrieved from the Israel Central Bureau of Statistics.

The study cohort was divided into two groups. The first group included patients with T2DM and gout arthropathy treated with DPP4 inhibitors, and the second group included patients with T2DM and gout arthropathy who were not treated with DPP4 inhibitors. Patients were considered to be treated with DPP4 inhibitors if there was documentation of at least four purchases of this medication.

### Eligibility criteria

Our cohort included patients with a diagnosis of T2DM and gout arthritis.

Inclusion criteria were patients with T2DM who were diagnosed with gout arthritis. Exclusion criteria were pregnant women and immunocompromised patients due to immunosuppressive drugs.

### Ethical approval

Ethical approval for this study was obtained from the Institutional Review Board of Rabin Medical Centre (RMC-0561-21). This manuscript adheres to the Strengthening the Reporting of Observational Studies in Epidemiology statement 21 .

### Measurements and data collection

The following data were collected from documentation by the family physicians and/or by referral and admission to the Emergency Room (ER): (1) Age, gender, year of diagnosis of T2DM, year of diagnosis of gout arthropathy, and number of annual gout flares, (2) Urate-lowering therapy (number of prescribed drugs and treatment duration) including allopurinol, febuxostat, anti-inflammatory drugs including colchicine, prednisone, and/or non-steroidal anti-inflammatory drugs, (3) Background comorbidities, including hypertension, dyslipidemia, smoking, myocardial infarction, and peripheral vascular disease, (4) Diabetic data included glycated hemoglobin (HbA1C), glucose levels, and anti-diabetic treatment, (5) Additional laboratory data included serum urate levels (before treatment and at follow-up) and hs-CRP.

### Definitions

Gout: Incident cases of gout arthritis were defined according to electronic health record (EHR) studies as described previously 22 23 . In short, gout arthritis was defined as follows: (1) International Classification of Diseases 10th version (ICD-10) codes M10.0 diagnosis from at least one rheumatologist visit, (2) ICD-10 M10.0 diagnosis or free text diagnosis of ‘gout’ from at least two community diagnoses at least 30 days apart between and either (a) the purchase of at least two gout-related prescription medications (allopurinol, probenecid, colchicine, or sulfinpyrazone) at least 30 days apart with the first within six months before or any time after the first community diagnosis or (b) two sUA test results>6 mg/dL with the first within 6 months before or any time after the first community diagnosis at least 30 days apart; (3) ICD-10 M10.0 diagnosis from at least one hospital admission diagnosis 24 .

Gout flare: Gout flares were defined as previously described 23 As follows: recorded hospital visit with gout (consultant or emergency visit or hospitalization) together with at least one of the following treatment patterns within 1 week: intra articular aspiration, intraarticular corticosteroid injection, prescription of non-steroidal anti-inflammatory drugs, or prescription of corticosteroids or adrenocorticotropic hormone (ACTH -Synacthen Depo).

T2DM: T2DM was defined as individuals who received diabetes treatment or had an ICD-10 diagnosis (E11) of diabetes mellitus as a chronic disease or reimbursements if at least three anti-diabetic drugs annually 24 .

CKD: CKD stage 3 was defined as moderate kidney damage with a reduced glomerular filtration rate of 30–59 mL/min/1.73 m² 25 .

### Statistical analysis

Descriptive statistics were used to summarise the data. The distribution of variables was visually assessed using histograms and QQ plots. Normally distributed numerical variables were presented as means±standard deviation (SD); non-normally distributed variables were presented as medians [25th to 75th percentiles]. Categorical variables were presented as frequency and percentages (%). Student t-test and chi-square were used to compare attack rates of gout arthritis over the one-year follow-up period between the two groups. Statistical analysis was performed using SAS, version 21.

## Results

Between 2016 and 2022, the study included 4,573 patients with gout and T2DM ( Table 1 ). Among these, 850 patients received DPP4 inhibitors, while 3,723 patients did not, serving as the control group. The average follow-up period was 102.35±69 months. The majority of patients were male, with a male-to-female ratio of 3.7:1. Notably, 75% of patients were diagnosed with gout before developing T2DM and had a high prevalence of metabolic syndrome. Specifically, essential hypertension was present in 3,359 patients (73.45%), dyslipidemia in 2,915 patients (63.74%), and the average BMI was 30.77±7.65 kg/m². Stage 3 CKD was diagnosed in 1,834 patients (40.1%).

**Table:** **Table 1** Baseline characteristics of study participants.This table presents the baseline demographic and clinical characteristics of the study population, including age, sex, BMI, comorbidities, medication use, and key laboratory values. Comparisons between the DPP-4 inhibitor group and the control group are shown, along with corresponding p-values. BMI: body mass index; DPP-4: dipeptidyl peptidase-4.

Variable	DPP4i treatment ^*^ (n=850)	Control (n=3723)	p-value
Age, years (mean±SD)	65.2±11.3	68.9±12.3	0.1
Male, n (%)	719 (78)	2938 (84)	0.8
T2DM, n (%)	197 (23)	904 (24)	0.6
Duration of T2DM -years (mean±SD)	10.9 + 2.8	10.2+1.9	0.75
HbA1C (%), (mean±SD)	7.3±1.2	7.4±1.1	0.25
Uric acid level (mg/dL) (mean±SD)	6.1 + 4.23	6.3 + 2.8	0.24
eGFR (mL/min/1.73 m ^2^ ) (mean±SD)	74 + 28	71 + 35	0.7
Hypertension, n (%)	616 (72)	2743 (73)	0.4
Dyslipidemia, n (%)	577 (67)	2338 (62)	0.07
BMI, kg/m ^2^ (mean±SD)	30.9±11.0	30.7±6.5	0.6
CKD, n (%)	262 (30)	1572 (42)	0.06
Current Smoking, n (%)	76 (8.9)	373 (10)	0.3
MI, n (%)	101 (15)	577 (11)	0.05
CVA, n (%)	15 (2)	123 (3)	0.09
PVD, n (%)	60 (7)	489 (12)	0.04

Categorical variables are presented as counts and percentages. Normally distributed continuous variables are presented as mean±standard deviation (SD). DPP4i treatment, defined as at least four purchases; T2DM, type 2 diabetes mellitus; HbA1C, hemoglobin A1c; BMI, body mass index; CKD, chronic kidney disease; MI, myocardial infarction; CVA, cerebrovascular attack; PVD, peripheral vascular disease; eGFR, estimated glomerular filtration rate; DPP-4i: dipeptidyl peptidase-4 inhibitor.

**Fig. 1:**
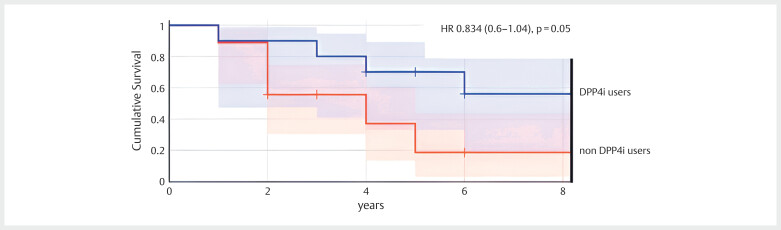
Kaplan-Meier survival curves for patients with CKD, T2DM, and gout treated with DPP-4 inhibitors vs. control group. The Kaplan-Meier survival curves illustrate the survival rates of patients with CKD, T2DM, and gout, stratified by treatment. The curves show a significant survival advantage for patients in the DPP-4 inhibitor group (solid line) compared to the control group (dashed line) over the follow-up period (mean±SD: 102.35±69 months). The HR for survival in the DPP-4 inhibitor group is 0.834 (95% CI: 0.6–1.04, p=0.05), indicating a trend toward improved survival, particularly in patients with CKD. CKD: chronic kidney disease; T2DM: type 2 diabetes mellitus; DPP4i: dipeptidyl peptidase-4 inhibitor; HR: hazard ratio.

The overall mortality rate was 15.67%, with no significant differences observed between the two groups. However, survival rates varied among patients with CKD. As shown in Fig. 1 , Kaplan-Meier survival analysis indicated that patients in the DPP-4 inhibitor treatment group had a hazard ratio (HR) of 0.834 (95% CI: 0.6–1.04, p=0.05), suggesting a trend toward more prolonged survival compared to the control group, although this association does not reach statistical significance.

**Fig. 2:**
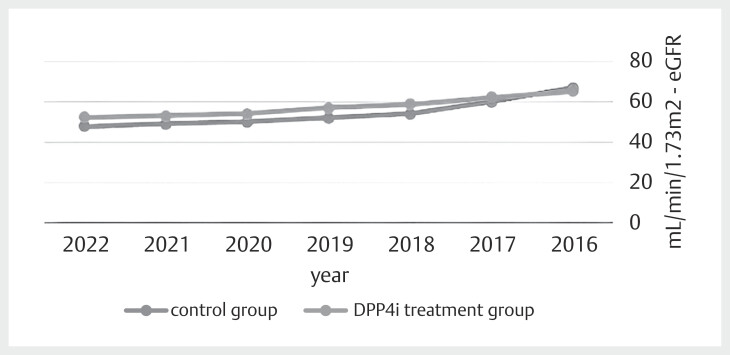
Trends of eGFR for patients with T2DM and gout treated with DPP-4 inhibitors vs. control group. This figure illustrates the trends in estimated glomerular filtration rate eGFR over the observation period for patients with T2DM and gout, comparing those treated with DPP-4 inhibitors to a control group. The eGFR values for the DPP-4 inhibitor group demonstrate a sustained advantage over time compared to the control group, indicating potentially better renal outcomes. The difference in eGFR between the groups is statistically significant, with patients in the DPP-4 inhibitor group showing a higher eGFR (p=0.042), suggesting that DPP-4 inhibitors may help preserve kidney function in this patient population. eGFR: estimated glomerular filtration rate; T2DM: type 2 diabetes mellitus; DPP4i: dipeptidyl peptidase-4 inhibitor.

Treatment with DPP4 inhibitors also reduced sUA levels (5.2±1.3 mg/dL vs. 5.9±2.2 mg/dL, p=0.05). Fig. 2 displays trends of eGFR for patients with T2DM and gout treated with DPP-4 inhibitors vs. control group. The curves show a significantly higher eGFR advantage for patients in the DPP-4 inhibitor group (p=0.042).

The frequency of ED visits due to gout attacks was initially similar between the groups but decreased during the follow-up period in patients treated with DPP4 inhibitors. Additionally, serum hs-CRP levels at baseline were comparable between the groups (0.86±0.33 mg/dL in the DPP4 inhibitor group vs. 0.90±0.29 mg/dL in the control group, p=0.09). However, hs-CRP levels significantly decreased during the follow-up in the DPP4 inhibitor group (0.50±0.19 mg/dL, p<0.001) Table 2 .

**Table:** **Table 2** Primary and secondary outcomes.This table summarises the primary and secondary study outcomes, including the frequency of gout flares, serum uric acid levels, hs-CRP levels, eGFR, and survival rates. Comparisons between the treatment and control groups are provided, along with statistical significance values. hs-CRP levels: high-sensitivity C-reactive protein; eGFR: estimated glomerular filtration rate.

Variable	DPP4i treatment (n=850)	Control (n=3723)	p-value
ER per year visits due to gout flares			
Pre-treatment ^*^	4.6 per year	4.4 per year	
Post-treatment	12 per year	84 per year	0.12
Uric acid, mg/dL (mean±SD)	5.2±1.3	5.9±2.2	0.05
Hs-CRP ^‡^ , mg/dL (mean±SD)			
Pre-treatment (mean±SD)	0.86±0.33	0.90±0.29	0.09
Post-treatment (mean±SD)	0.50±0.19	0.90±0.29	<0.001

Normally distributed continuous variables are presented as mean±SD. ^*^ Pre-treatment, before the first DPP4 inhibitor purchase ^†^ ER, emergency room; ^‡^ Hs-CRP, high sensitivity C-reactive protein. DPP4i: dipeptidyl peptidase-4 inhibitor.

## Discussion

In this population-based, longitudinal study, we investigated the effect of DPP4i treatment on the frequency of gout arthritis in patients with T2DM and the impact on CKD. Our study found that treatment with DPP4i resulted in a decrease in ED visits due to gout flares. Despite a lack of statistical significance, the reduction in ER visits from 28 to 12 is still clinically significant.

While treatment with DPP4 inhibitors did not affect the mortality rate in the study groups, it significantly affected the survival rates among those patients with CKD who were diagnosed with T2DM and gout. In a trial Evaluating Cardiovascular Outcomes with Sitagliptin (TECOS) 26 , including over 14,000 patients with T2DM and a history of cardiovascular disease, sitagliptin was associated with a lower risk of all-cause mortality compared to placebo over a median follow-up period of 3 years. In agreement with previous studies 26 27 28 , our study shows beneficial effects on survival in diabetic patients with CKD treated with DPP4 inhibitors.

Our study also demonstrated a reduction in sUA levels in the DPP-4 inhibitor group. While previous studies have shown that DPP-4 inhibitors may lower sUA levels through mechanisms such as reducing xanthine dehydrogenase expression 29 30 , another possible explanation is the better preservation of renal function in the DPP-4 inhibitor group. As demonstrated in Fig. 2 , patients treated with DPP-4 inhibitors exhibited significantly higher eGFR levels compared to the control group (p=0.042). Since the kidneys play a primary role in uric acid excretion, improved renal function could have facilitated better uric acid clearance, contributing to the observed reduction in sUA levels. This aligns with existing literature suggesting that DPP-4 inhibitors have renoprotective effects, including reduced albuminuria and anti-inflammatory benefits, 19 27 which may slow CKD progression.

Further, a post hoc analysis of a randomized controlled trial comprised of 7,928 patients with T2DM found a lower risk of developing gout in those patients who received the DPP4i linagliptin 31 .

The most common causes of mortality in patients with gout include cardiovascular disease, CKD, and infections 32 33 34 . In patients with gout and CKD, the latter is a significant cause of mortality, which may be related to both the effects of hyperuricemia on the kidneys and the development of metabolic syndrome 33 34 .

Here, we found that DPP4 inhibitor treatment decreased sUA levels and improved survival rates in patients with T2DM and CKD ( Fig. 1 ). DPP4 inhibitors may have renal protective effects. Studies have shown that DPP4 inhibitors can reduce urinary albumin excretion, a marker of kidney damage, in patients with T2DM and early-stage diabetic nephropathy 27 .

Furthermore, our study demonstrated a significant reduction in serum hs-CRP levels among patients treated with DPP4 inhibitor therapy, suggesting an anti-inflammatory effect. Indeed, treatment of T2DM patients with the DPP4i linagliptin was shown to be associated with a breakdown and reduction of several inflammatory peptides, including incretins, neuropeptide Y, and substance P 19 .

A network meta-analysis, including DPP4 inhibitors among other newer glucose-lowering drugs, found no reduction in gout flares among patients treated with DPP4 inhibitors. Our study found a tendency towards a reduction in gout flares, although insignificant.

### Limitations

This study has several limitations. First, the retrospective nature of the study design introduces the possibility of selection bias, as patient data were derived from EHR, which may be incomplete or inaccurate. Second, while our study included all patients with T2DM and gout regardless of their antidiabetic treatment regimen, this may have introduced some heterogeneity within the control group, particularly if a higher proportion of insulin users was included due to advanced disease progression. The significantly higher prevalence of peripheral vascular disease in the control group (12% vs. 7%, p=0.04) supports this possibility. However, HbA1c levels were comparable between the groups (DPP-4 inhibitor group: 7.3±1.2% vs. Control: 7.4±1.1%, p=0.28), with no significant difference, suggesting that glycemic control at baseline was similar and minimizing the impact of diabetes severity on our findings. Additionally, since our primary endpoints—sUA levels, hs-CRP reduction, and survival trends—are independent of short-term glycemic fluctuations, the lack of stratification by diabetes treatment does not significantly affect the validity of our conclusions. Third, the small statistical difference may be a chance finding due to the study's group sizes and the retrospective nature of the study. Finally, the study did not include a direct comparison of different DPP-4 inhibitors, which could have provided insights into the relative efficacy of each medication.

## Conclusion

Our findings suggest that DPP-4 inhibitors may provide benefits beyond glycemic control in patients with T2DM, gout, and CKD. Specifically, treatment with DPP-4 inhibitors was associated with lower sUA levels, reduced hs-CRP levels, and improved renal function. Additionally, there was a trend towards improved survival among patients with CKD. While these findings are promising, the retrospective nature of the study and the observed heterogeneity in treatment regimens warrant further investigation. Future prospective and randomized controlled trials are necessary to confirm these results and explore the potential anti-inflammatory and renoprotective mechanisms of DPP-4 inhibitors in this patient population.
